# SQUAT: a Sequencing Quality Assessment Tool for data quality assessments of genome assemblies

**DOI:** 10.1186/s12864-019-5445-3

**Published:** 2019-04-18

**Authors:** Li-An Yang, Yu-Jung Chang, Shu-Hwa Chen, Chung-Yen Lin, Jan-Ming Ho

**Affiliations:** 1 Institute of Information Science, Academia Sinica, Taipei, Taiwan; 2 Research Center for Information Technology Innovation, Academia Sinica, Taipei, Taiwan

**Keywords:** Data quality assessment, Genome assembly, Genome sequencing, Non-model organisms

## Abstract

**Background:**

With the rapid increase in genome sequencing projects for non-model organisms, numerous genome assemblies are currently in progress or available as drafts, but not made available as satisfactory, usable genomes. Data quality assessment of genome assemblies is gaining importance not only for people who perform the assembly/re-assembly processes, but also for those who attempt to use assemblies as maps in downstream analyses. Recent studies of the quality control, quality evaluation/ assessment of genome assemblies have focused on either quality control of reads before assemblies or evaluation of the assemblies with respect to their contiguity and correctness. However, correctness assessment depends on a reference and is not applicable for de novo assembly projects. Hence, development of methods providing both post-assembly and pre-assembly quality assessment reports for examining the quality/correctness of de novo assemblies and the input reads is worth studying.

**Results:**

We present SQUAT, an efficient tool for both pre-assembly and post-assembly quality assessment of de novo genome assemblies. The pre-assembly module of SQUAT computes quality statistics of reads and presents the analysis in a well-designed interface to visualize the distribution of high- and poor-quality reads in a portable HTML report. The post-assembly module of SQUAT provides read mapping analytics in an HTML format. We categorized reads into several groups including uniquely mapped reads, multiply mapped, unmapped reads; for uniquely mapped reads, we further categorized them into perfectly matched, with substitutions, containing clips, and the others. We carefully defined the poorly mapped (PM) reads into several groups to prevent the underestimation of unmapped reads; indeed, a high PM% would be a sign of a poor assembly that requires researchers’ attention for further examination or improvements before using the assembly. Finally, we evaluate SQUAT with six datasets, including the genome assemblies for eel, worm, mushroom, and three bacteria. The results show that SQUAT reports provide useful information with details for assessing the quality of assemblies and reads.

**Availability:**

The SQUAT software with links to both its docker image and the on-line manual is freely available at https://github.com/luke831215/SQUAT.

**Electronic supplementary material:**

The online version of this article (10.1186/s12864-019-5445-3) contains supplementary material, which is available to authorized users.

## Background

The ultra-high throughput provided at low cost by recent next-generation sequencing technologies has triggered the rapid growth of whole-genome sequencing projects, especially for non-model organisms [1, 2]. Large-scale genome projects for broad taxa, such as the Genome 10 K Project for vertebrate species [3], the Global Invertebrate Genomics Alliance (GIGA) for marine invertebrate species [4] and the latest Earth BioGenome Project that aims to sequence genomes of ~ 1.5 million known eukaryotic species over a 10-year period [5], have brought new challenges in assembling and analyzing the forthcoming de novo genome assemblies. One important challenge is regulating the quality of sequencing data and assembly results.

Data quality assessment (DQA) of genome assemblies is a process to statistically evaluate the input data and the assembly results and then determine whether the data and assembly results meet the quality requirements. DQA is an important task for de novo genome assembly and is especially useful today, as massive genome assemblies are in progress or available as drafts. Recent studies on the quality related issues of genome assemblies have focused on two aspects: quality control of sequencing data and quality assessment of assembly results. For the Illumina platform, FastQC [6] provides quality control checks in an HTML report that includes per-base quality, average read quality, GC content, sequence length, duplication levels, and overrepresented sequences. NGS QC Toolkit [7] provides various tools, including quality control, trimming, format conversion, and statistics, for quality check and filtering of high-quality data. QC-chain [8] focuses on quality assessment and trimming of raw reads, as well as identification and filtration of unknown contamination. ClinQC [9] integrates several QC tools for clinical purposes. For quality evaluation/assessment of assembly results, GAGE [10] and GAGE-B [11] provide benchmark datasets along with functions such as those evaluating the correctness of assemblies if the reference genomes are given. QUAST [12] is a quality assessment tool for evaluating and comparing genome assemblies. It provides comprehensive metrics of assembly contiguity, i.e. the length statistics of scaffolds, in HTML reports for de novo assemblies, and supports a GAGE mode if the references are available.

The SQUAT tool aims to provide quality assessments for both genome assemblies and their input reads, and helps users to examine the correctness of de novo assemblies via cross-checking both the pre-assembly and post-assembly reports. The pre-assembly module of SQUAT computes quality statistics of sequencing reads and presents the analysis results in a well-designed interactive HTML interface. Meanwhile, we divide reads into three groups, i.e., high-, medium- and poor-quality reads for overall assessment. The classification criteria are based on the MinimalQ measure for read subset selection [13] and a new measure by generalization of MinimalQ described in Methods. The post-assembly module of SQUAT performs read mapping and classifies reads into several groups based on read-to-scaffold relationship; further, it assists users in identifying poorly mapped (PM) reads by including not only unmapped reads, but also reads with high clip ratio or high mismatch ratio via further analyses described in Methods. We present the tables and charts of the aforementioned classification and analyses, integrated with QUAST results, in well-designed HTML interface. In the Results, we have evaluated SQUAT against six datasets. Eel, mushroom, and worm datasets are from de novo genome projects in progress, which are being led by our institute. We also analyzed three bacterial datasets listed in the SPAdes’s GAGE-B report [14, 15]. The results show that SQUAT can successfully differentiate the quality of those datasets, even though we only used one million randomly-sampled reads. In addition, the results can help to explain why some datasets have serious clips and/or mismatches in the reads.

## Methods

### The SQUAT assessment procedure

**Fig. 1:**
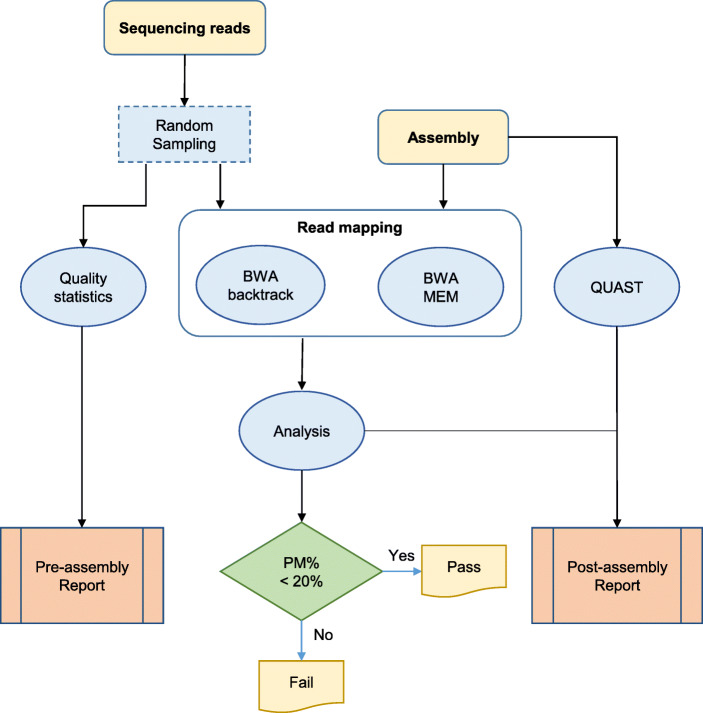
SQUAT assessment workflow

The workflow of SQUAT assessment is shown in Fig. 1. The whole process takes sequencing reads and their assembly as input and generates both pre-assembly and post-assembly HTML reports to help users examine their data from different perspectives. To begin with, we randomly sample one million entries of reads from the original dataset for a quick examination. Note that users can change the default sample size of ‘one mega’ or bypass the sampling process, as shown in the manual. The pre-assembly workflow is shown on the left side of Fig. 1. The quality statistics module takes the sampled reads as input and generates tables and distributions in the HTML report for evaluating the base quality and read quality in detail. It also presents a pie chart on top of the report to show the proportions of poor-, medium- and high-quality reads for overall assessments.

**Fig. 2:**
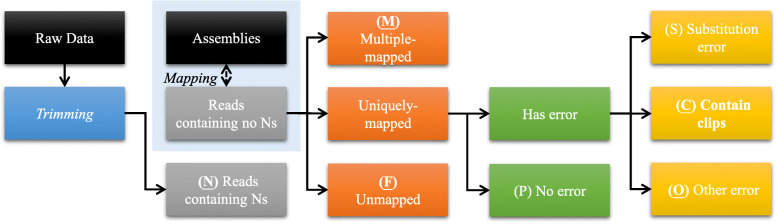
Classification of reads by read mapping analysis. The descriptions and icons of these read labels are shown in Table 1

The post-assembly workflow, depicted in the middle and right parts of Fig. 1, firstly maps the sampled reads onto the input scaffolds of genome assembly by a local aligner BWA backtrack [16] and an end-to-end aligner BWA MEM [17]. Then, the Analysis module 1) categorizes the reads into seven groups, as shown in Fig. 2 and Table 1 (will be described later in the sub-section Post-assembly analysis), and 2) generates the percentage of poorly-mapped reads by considering not only unmapped reads, but also reads with abnormal densities of substitutions and clips.

**Table 1:**
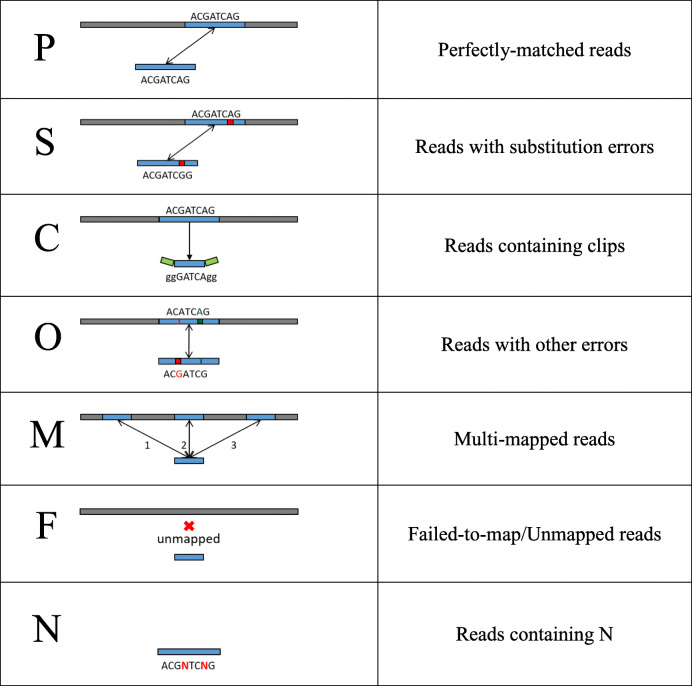
Summary of post-assembly read label tagging with icons

### Pre-assembly analysis

The pre-assembly report assesses sequencing reads based on base quality scores. We analyze the sequencing reads of the dataset by computing the base quality scores of each read. According to Fang’s work [13], reads with low minimal quality values (MinimalQ) are more likely to cause mis-assemblies. Therefore, we identify the MinimalQ, i.e., the minimal quality score of bases of each read to get a sense of the sequencing quality.

The flow of the pre-assembly analysis is as follows. First, we compute the basic statistics of the input FASTQ file, including numbers of bases and reads, min/max/average length of reads, frequencies of DNA alphabets and distribution of GC contents of the reads. Then we compute the quality statistics, including distribution of base quality scores, distribution of reads’ MinimalQ values, and cumulative distribution of reads’ high-quality portions. We define %HighQ(*q*) for a read as follows:

$$ \%\mathrm{HighQ}(q)=\frac{number\ of\ bases\ with\ qual\mathrm{i} ty\  scores\ge q}{number\ of\  all\  bases} $$

For example, if a set of reads satisfies the equation *%HighQ(20) = 100%*, then for each read of the set, 100% of the bases have quality scores of Q20 & above, which is equivalent to *MinimalQ ≥ 20*. We can also find reads satisfying the equation *%HighQ(15) ≥ 90%*, i.e., more than 90% of their bases with Q15 & above, to select reads with better-than-poor quality.

To get a summary of the quality statistics, we categorize reads into three groups. The categorization process of poor-, medium- and high-quality reads is as follows: First, reads having every base scored Q20 & above are labeled as high-quality and the classification criterion is based on the MinimalQ measure for read subset selection [13]. Then, we label reads with more than 10% of bases having quality scores less than Q15 as poor-quality reads. Reads falling into neither category are labelled as medium-quality. Finally, we visualize the distributions of basic statistics, quality statistics and the categorization results in a well-designed HTML format as shown in Additional file 1.

### Post-assembly analysis

For the post-assembly report, we ask users to input the path of a sequencing read file and its assembly as a reference, and map them to the assembly twice using different alignment algorithms: BWA-MEM and BWA-backtrack. After the mapping process, we extract alignment information from the generated SAM file to perform detailed analysis and compute the percentage of poorly-mapped reads (PM%). By default, the dataset will pass the assessment if it contains less than 20% of poorly-mapped reads (PM% < 20%). We integrate our results and analysis with the assembly evaluation by QUAST into several tables and figures in the report. The rest of this section illustrates how each step of post-assembly analysis works in detail.

#### Read label tagging

The designated process of read label tagging during the phase of read mapping is shown in Fig. 2. We first screen out the reads containing *N*s (i.e., ambiguous result of base-calling) and label them with type **N**. Then, the rest of the reads that have no *N*s will be labeled by read mapping. The mapping step of our proposed procedure aims to inspect the similarity between reads and their corresponding location on the assembly and reports the alignment information in SAM format. After finishing this step, reads are tagged with six other types of labels alongside type **N**. First, for the reads that cannot be mapped to the assembly (i.e., failed to map), a type of **F** is tagged.

The remaining untagged reads are the reads that are mapped to the assembly at least once. The reads that occur in multiple locations, called *repeats*, are labeled as type **M**. For the *unique* reads (i.e., reads that occur once on the assembly), we then check if the mapping includes any error. The alignment without any errors is a perfect match and labeled as type P. For those with at least one substitution error, a type of S is tagged. Otherwise, if a read contains clips on either side of the alignment, it will be labeled as type C. The rest of the reads, which normally contain insertion or deletion errors, are then assigned to type O (others). For the sake of posterior lookup, we summarize these read labels with icons and descriptions in Table 1. The detailed classification flow is given in Additional file 2.

#### Alignment algorithms

For read mapping, we adopt two strategies: BWA-MEM and BWA-backtrack. BWA-MEM performs local alignment and may produce multiple alignments for a different part of a query sequence. It is designed for longer sequences ranging from 70 bp to 1 Mbp. On the other hand, BWA-backtrack is more suitable for short reads because it tries to map the whole sequence (end to end strategy). However, the latter algorithm cannot tolerate as many sequencing errors as the former.

#### Analysis modules

**Fig. 3:**
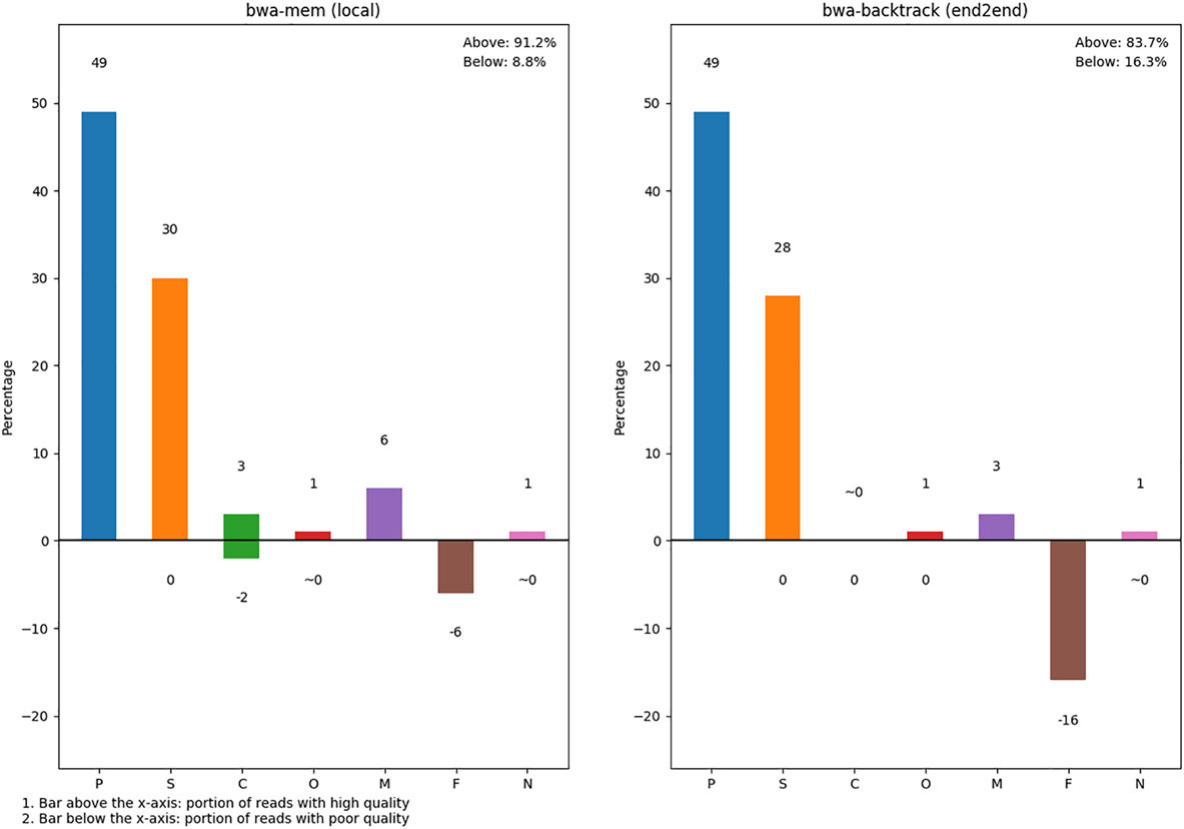
Label distribution barchart of the mushroom dataset. The poorly-mapped ratio (PM%) of Mushroom dataset is 8.8% on the left with BWA-MEM and 16.3% on the right with BWA-backtrack

The analysis modules used by SQUAT operate upon different read labels in an attempt to differentiate poorly-mapped reads from the good ones. To construct an overview of read mapping quality, we plot a label distribution bar chart to summarize the overall alignment condition in one place, as shown in Fig. 3. To begin with, perfectly-matched (type P) and multi-mapped reads (type M) are considered highly-mapped, while unmapped reads (type F) are poorly-mapped. Next, on top of the label distribution derived from the process of read label tagging, we derive three metrics, mismatch ratio, clip ratio, and N ratio to represent the percentage of poorly-mapped segments in sequences tagged as S, C, and N respectively. The clip ratio, for example, is computed as follows:

$$ \mathrm{Clip}\ \mathrm{ratio}=\mathrm{total}\ \mathrm{length}\ \mathrm{of}\ \mathrm{clips}/\mathrm{read}\ \mathrm{length} $$

**Fig. 4:**
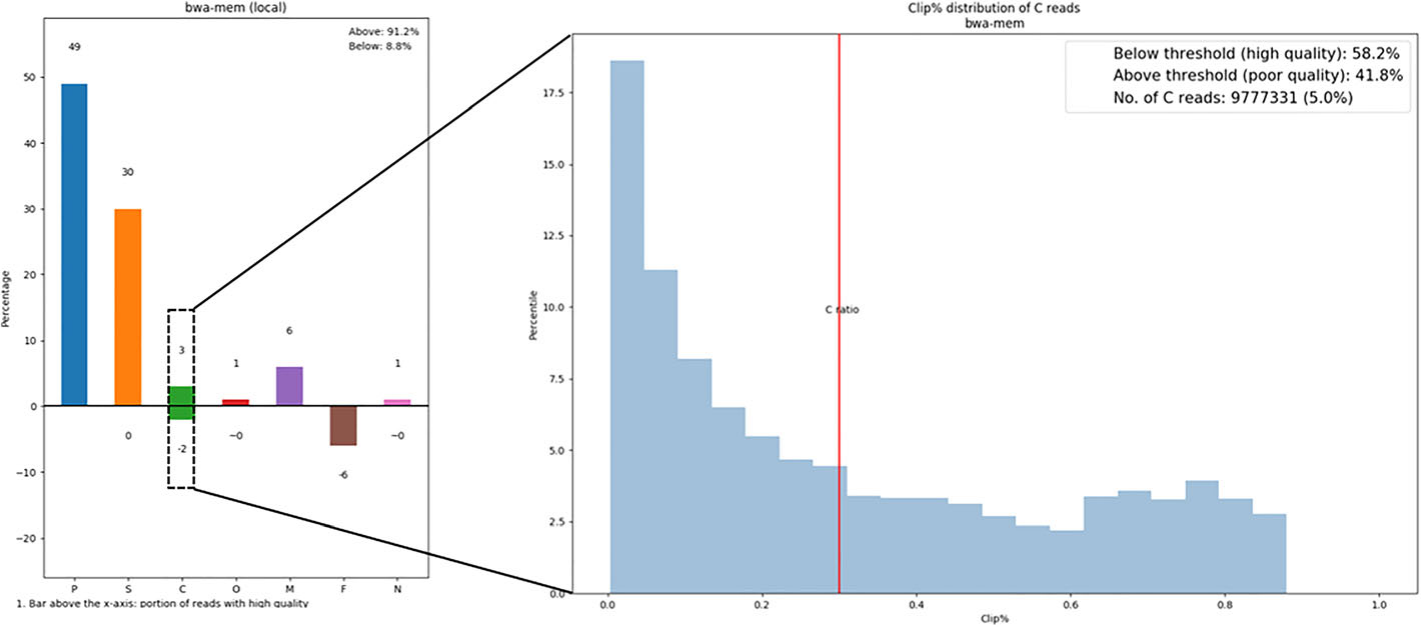
Clip ratio distribution of the mushroom dataset. The threshold value is set at 0.3 by default

We then specify a threshold to partition the clipped reads into two groups. For example, the threshold value for the clip ratio distribution of Mushroom in Fig. 4 is set at 0.3, and the reads distributed on the right of the threshold are identified as poorly mapped, while the other half are high-quality reads whose clip ratios are below the threshold.

As for the bar chart, we use bars of positive and negative values to represent the percentage of high and poor mapping quality reads accordingly. Thus, the sum of the negative values in a bar chart is defined as the poorly-mapped ratio (PM%) of the dataset. Because two alignment algorithms are used to manipulate two PM% values, we average the two statistics to obtain the final PM%. For the Mushroom dataset, the final average PM% is 12.5%.

#### Alignment score

**Fig. 5:**
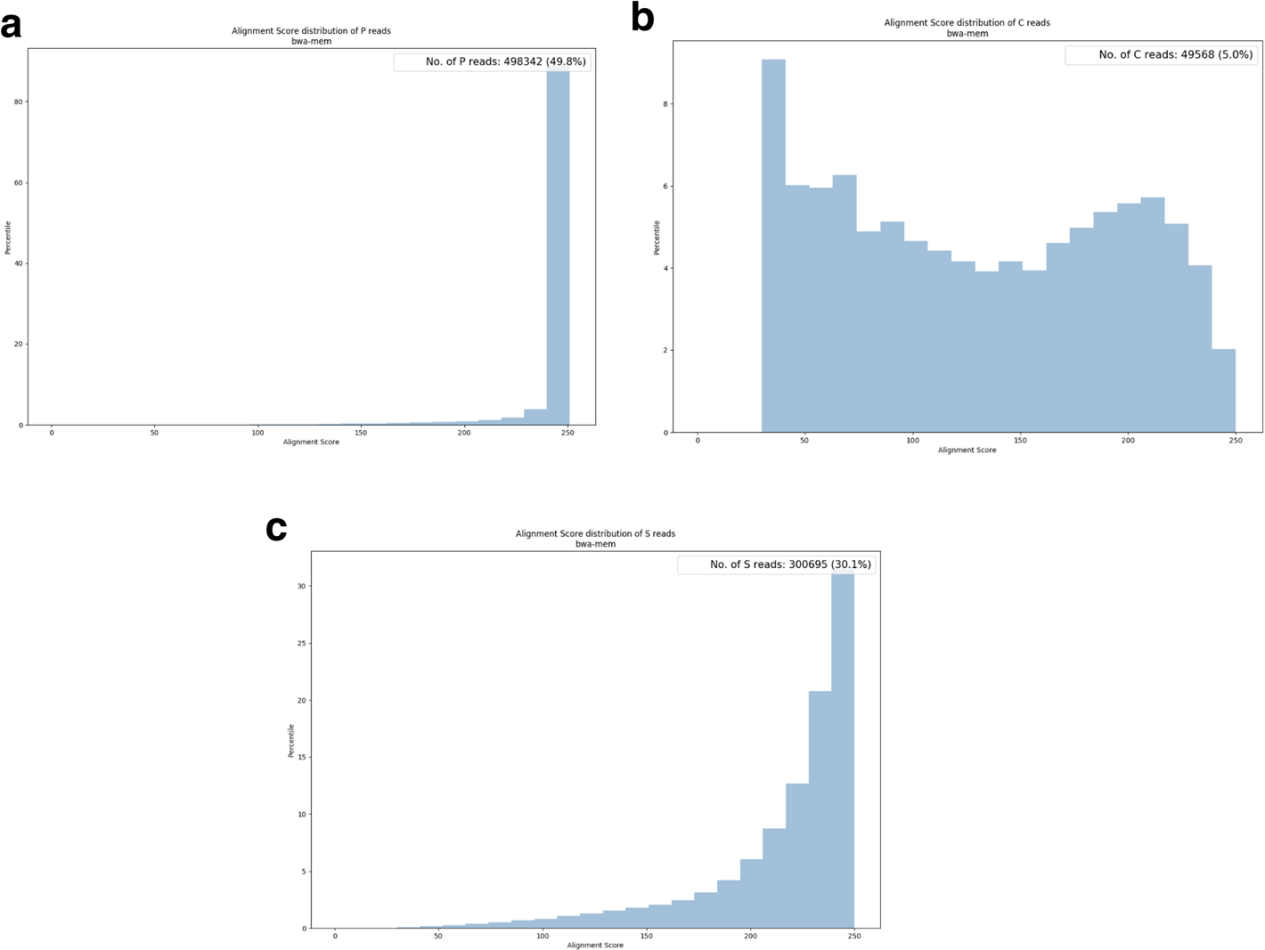
Alignment score distribution for the mushroom dataset. **a** Distribution of reads with no errors (type P). **b** Distribution of reads with substitution errors (type S). **c** Distribution of reads containing clips (type C). The majority of the alignment scores of reads goes from P, S, to C in decreasing order

For each alignment, BWA also records an alignment score to represent the similarity between a read and its mapped area on the assembly. The score increases with the number of matches and is penalized by the number of mismatches and gaps. Therefore, a higher alignment score implies a better alignment result. We extract alignment scores from the SAM file and plot the distribution for P, S, and C reads in the report, as in Fig. 5. The median values of the alignment score decline from P to S to C.

## Results

### Datasets

We evaluate SQUAT with six datasets, three of which are generated by next-generation sequencers. They belong to the realm of de novo genome assembly and are denoted as Mushroom, Eel, and Worm. For Eel and Worm, we further divide the data according to the insert size. The name of each dataset is therefore followed by the corresponding insert size, e.g., Worm 1300. The other three are bacterial datasets from GAGE-B [11, 15], denoted as D1, D2, and D3. The profile of our experimental dataset is shown in Table 2. We also incorporate a trimming step by TrimGalore [18] to remove adapters, vectors, or primers used in these datasets.

**Table 2:** The sequencing datasets used in the experiment

Dataset	Mushroom	Eel	Worm	D1	D2	D3
Scientific Name	*Termitomyces eurhizus*	*Anguilla japonica*	*Aeolosoma Viride*	*R. Sphaeroides*	*M. abscessus*	*V. cholerae*
Accession number	unpublished	*PRJEB25708*	unpublished	*SRR522246*	*SRA043447*	*SRA037376*
# reads (M)	196.8	306.3	98.2	16.3	8.7	7.0
Assembly size (Mbp)	77.0	1022.0	741	4.6	5.6	5.0
Sequencing depth	~ 250	~ 160	~ 230	245.2	194.3	195.7
Read length	25–251	35–201	35–301	25–251	25–251	25–251
Reference Genome (NCBI accession number)	N/A	N/A	N/A	*R.sphaeroides 2.4.1*	*M.abscessus ATCC 19977*	*V.cholerae 01 biovar eltor str. N16961*

### Implementation resources

In the experiment, we incorporate BWA [16, 17] (version 0.7.15) for mapping sequences to their assembly or reference genome and generate a SAM [19] file following each process. To assemble the reads of the three bacteria in Table 2, we use SPAdes [14] (version 3.11.1). Tools for the assemblies of eel, worm, and mushroom include ALLPATHS-LG [20], SSPACE [21] and GapCloser [22]. Finally, we use QUAST [12] (version 4.6.3) to evaluate genome assemblies with various metrics such as

- Number of scaffolds
- Length of Max/N25/N50/N75/L80/L90/L99 scaffold
- Number of unknown base N’s per 100 kbp
- GC percentage

### Pre-assembly statistics

The results of pre-assembly quality assessments for each dataset are summarized in Table 3. For D1 dataset, the percentage of bases with Q30 & above is 40.4%, which is the lowest in Table 3; meanwhile, the percentages of poor- and high-quality reads are 96.1 and 1.5%, respectively.

**Table 3:** Summary of the pre-assembly reports for each dataset

Measure %	Mush -room	Eel 1300^a^	Eel 500	Eel 400	Worm 1300	Worm 620	D1	D2	D3
% of bases with Q30^b^	89.4	88.0	93.6	92.6	68.6	87.6	40.4	69.0	83.6
% of high-quality reads^c^	21.9	51.4	66.5	60.7	0.1	3.2	1.5	14.6	30.6
% of medium-quality reads^d^	68.5	42.0	31.4	36.9	95.0	93.7	2.4	49.5	51.6
% of poor-quality reads^e^	9.6	6.6	2.1	2.4	4.9	3.1	96.1	35.9	17.8

^a^The number indicates the insert size of the specie

^b^The percentage of bases with quality values ≥ 30

^c^The percentage of reads that all of their base with Q20 & above

^d^The percentage of reads that are not high-quality or poor-quality

^e^The percentage of reads that more than 10% of their bases with Q14 or less

### Post-assembly report interface

**Fig. 6:**
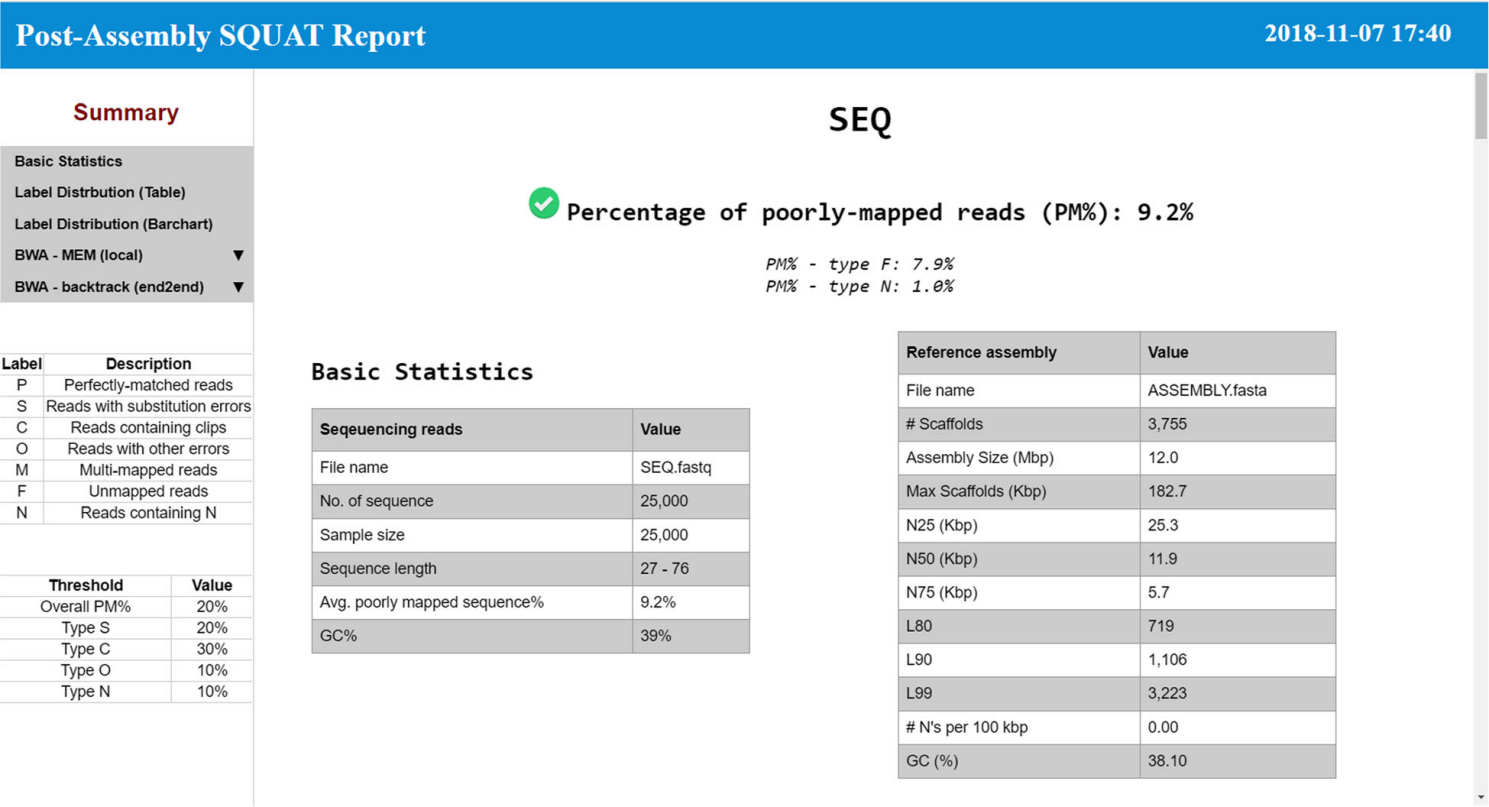
Post-assembly report interface

Figure 6 displays a screenshot of the post-assembly report interface. We first show the percentage of poorly-mapped reads (PM%) as a summary statistic. Subsequently, the basic statistics of sequencing data and assembly are listed. For sequencing reads, we record information, such as number of sequences, sample size, and sequence length. For the assembly, we compute number of scaffolds, assembly size, N50 scaffold length, and several other evaluation metrics computed by QUAST.

On the left side of the report, we also include the description for each read label and specify the threshold values that determine the sequencing quality as reference for users while they scroll down the webpage to look for further analysis.

In summary, the report will lay out two tables and nine figures to perform complete analysis including label distribution, mismatch ratio, clip ratio, alignment score, and an overall PM% based on BWA-MEM and BWA-backtrack. Examples of post-assembly HTML reports are given in Additional file 3.

### Post-assembly statistics

The profiles of read label for all the datasets based on BWA-MEM are shown in Table 4. Note that there is no reference genome in a de novo assembly; thus, here we use scaffolds assembled from the reads as reference for read mapping. Take Mushroom for example; out of the 197 million reads, 12.5% of reads are considered poorly-mapped and 49.8% are classified as perfectly-matched (type P), while 30.1% contain substitution errors (type S). For the rest of the uniquely-mapped reads, 5.0% contain clips (type C), and 1.3% have other errors (type O). Another 6.6% of reads are tagged as unmapped (type F), 6.1% of reads are multi-mapped (type M), and 1.2% of reads contain Ns (type N).

**Table 4:** Profile of tagged labels and PM% for each dataset

reads %^a^	Mushroom	Eel 1300^b^	Eel 500	Eel 400	Worm 1300	Worm 620	D1	D2	D3
P	49.8	39.3	34.9	33.4	6.8	17.6	3.5	54.5	71.1
S	30.1	28.7	33.1	34.7	44.5	41.0	8.5	28.4	22.6
C	5.0	3.7	6.1	6.0	12.0	10.4	81.2	15.9	5.4
O	1.3	3.8	8.3	8.1	4.8	6.6	0.2	0.1	0.2
M	6.1	14.2	10.1	10.4	11.1	11.0	0.5	0.0	0.1
F	6.6	6.9	6.9	6.9	20.8	13.1	4.9	0.9	0.5
N	1.2	3.5	0.1	0.1	0.0	0.2	1.3	0.2	0.2
PM%	12.5	12.0	14.9	15.0	35.9	26.1	59	11.4	4.3

^a^The profile is based on BWA-MEM algorithm for greater tolerance in varied read length except PM%. PM% is based on the average score of BWA MEM and BWA backtrack, and considers both the fail-to-map reads and low-score clip reads as shown in Methods

^b^The number indicates the insert size of the specie

Of all datasets, D3 holds the lowest PM% (4.3%) and the highest percentage of reads with a perfect match (type P), representing over 70% of reads. On the other hand, Worm 1300 and D1 are estimated to have the highest PM% at 35.9 and 59%, respectively.

Worm 1300 contains the largest portion of reads with substitution errors (type S) and failed-to-map reads (type F) compared with other species. The signature of a low PM% can also be observed through its assembly quality. As shown in Table 5, the N50 scaffold size of Worm is far worse than the other two de novo genome assemblies. The relatively poorly-assembled scaffolds have allowed more than 20% of unmapped reads, as well as 44.5% of reads with substitution errors. As for D1, its inferior PM% could result from the fact that it consists of mostly reads containing clips (type C), at 81.2%, rendering the reads poor in mapping quality. But D1 has the highest sequencing depth than D2 and D3 (Table 2). Table 6 shows the assembly evaluation results of D1, D2 and D3.

**Table 5:** Assembly evaluation of the de novo assembly datasets

Dataset^a^	Mushroom	Eel	Worm
#Scaffolds^b^	800	7804	7280
Assembly size (Mbp)	77	1022	740
Max Scaffolds (Kbp)	2357	14,119	1562
N25 (Kbp)	959	4137	239
N50 (Kbp)	587	2148	138
N75 (Kbp)	293	954	78
L99	453	3474	6995
GC%	46.34	42.37	31.43

^a^These de novo assembly datasets don’t normally have reference genomes

^b^The scaffolds are assembled by All-paths LG and evaluated by QUAST

**Table 6:** Assembly evaluation of the three GAGE-B datasets

Dataset^a^	D1	D2	D3
#Scaffolds	109	912	1848
Assembly size (Mbp)	4.6	5.6	5.0
N50 (Kbp)	552	313	350
Max Scaffolds (Kbp)	1233	1346	737
NG25 (Kbp)	1233	1346	555
NG50 (Kbp)	552	313	356
NG75 (Kbp)	204	173	232
LG99	22	23	80
GC%	68.66	61.65	44.66
Indels ≥5	7	10	6
Inversions	0	0	0
Relocation	4	2	4
Translocation	1	0	1

^a^The scaffolds are assembled by SPAdes and evaluated by QUAST in GAGE mode

### Combined analysis of both pre- and post-assembly statistics

By looking at the D1 column in both Table 4 and 3, we found the D1 dataset contains 81.2% of type **C**, 8.5% of type **S** and 4.9% of type **F**, where the sum is 94.6%. Meanwhile, D1’s percentage of poor-quality reads is 96.1%. Thus, the 96.1% of poor-quality reads in the pre-assembly report can explain why D1 has such highly clipped reads.

In addition, the combined analysis may help to take proper actions to improve assemblies. Take the datasets with PM% > 20% in Tables 3 and 4 as examples, D1 dataset has a high percentage of poor-quality reads and clipped reads, and it may need to sequence the genome again to improve the read quality. A low percentage of high-quality reads and perfectly-mapped reads (e.g., the two worm datasets), it may need to sequence more reads with better quality.

### Time complexity and performance

SQUAT has four major modules, including random sampling, read quality analysis, read mapping and mapping analyzer. We also integrate QUAST in SQUAT. The time complexity of the random sampling is O(fastq_size + sampled_fastq_size). The read quality analysis costs O(sampled_fastq_size). For example, for a wheat fastq file (SRR5815659_1) containing 394 million 150-bp reads, where the total bases are 59 Gbp and the file size is 152 GB, the random sampling took ~ 40 min and the read quality analysis took ~ 29 min to process the whole fastq file on a virtual machine with one core and 2 GB RAM. For the one million sampled reads, the read quality analysis took ~ 3 s. The runtime of read mapping module that uses both BWA MEM and BWA backtrack is related to the sampled fastq size, the assembly size and the number of read hits in the sam file (denoted as #read_hit_in_sam). The actual runtime can be a few tens of minutes to hours, depending on the portion of repeat regions in genomes and the genome size. The time complexity of mapping analysis is O(#read_hit_in_sam). SQUAT also supports multi-threading in BWA alignment and QUAST evaluation. For example, SQUAT took ~ 12.6 min for the D1 dataset and ~ 22 min for the D2 dataset using two cores and ~ 2.3 GB RAM, but it took ~ 10 h using 32 cores and ~ 14.3 GB RAM for the wheat fastq because the wheat genome assembly is large, hexaploid, and highly repetitive. The details of the runtime experiments are given in Additional file 4.

### Comparison of main features with other tools for quality assessment of de novo assembly and sequencing data

First, we compare the main features of SQUAT with other tools for quality assessment/evaluation of de novo assembly in Table 7, including QUAST [12], REAPR [23] and BUSCO [24]. Each aforementioned tool has its own unique features and solves different problems. QUAST can be used with or without a reference genome. For de novo assemblies, QUAST provides comprehensive metrics and plots for evaluating assembly contiguity beyond N50, including N*x* plot, GC plot and cumulative plot of largest contigs. Meanwhile, users can compare multiple assemblies in a unified HTML interface. REAPR maps reads to assemblies and performs base-by-base analysis to compute fragment coverage distribution (FCD). It then uses FCD to identify scaffolding errors for improving assembly correctness. BUSCO analyzes the coverage of single-copy orthologues to evaluate assembly completeness. SQUAT focuses on the two-way quality assessment of assemblies and sequencing reads for examine assembly quality/correctness and provides detailed graphical reports to aid in tracing the reasons for poor assembly results. SQUAT also integrates QUAST into the reports.

**Table 7:** Comparison of main features with other tools for quality assessment of de novo assembly

Feature\Tools	SQUAT	QUAST	REAPR	BUSCO
Categorization of read-to-assembly mapping relationship	√			
Distributions of alignment scores, mismatch ratios, and clip ratios of read mapping results	√			
Evaluation of assembly quality by identification of poorly/properly mapped reads of different types	√			
Comprehensive metrics for evaluating assembly contiguity. Nx-like plots and cumulative plots		√		
Comparison of multiple assemblies		√		
Identification of scaffolding error by fragment coverage distribution			√	
Evaluation of assembly completeness by single-copy orthologues				√
Built-in light-weight assessment of read quality by categorizing reads into poor−/medium−/high- quality groups	√			

The pre-assembly quality assessment procedure is lightweight, and its reports are mainly for cross-checking with post-assembly reports. When comparing various features with other QC tools as listed in the ClinQC [9] paper, which has 20 features in a table, most of the features are not included in SQUAT, except three features, including virtual machine (we provide a docker image), graphical QC report, and GC content assessment. However, SQUAT’s pre-assembly analysis is lightweight and fast because it is written in C++ and can process 1 million 150-reads in a few seconds with ~ 3 MB memory. Its quality metrics come from a generalization of the MinimalQ method [13], which is useful for selecting high-quality read subsets to improve genome assemblies of high-depth NGS data. The generalized programs of pre-assembly read subset selection are written in C++ and included in SQUAT’s GitHub project (at library/preQ/). In addition, users can combine the read categorization results from both pre-assembly and post-assembly reports to evaluate assemblies with further actions as mentioned in the section of combined analysis.

## Discussion

In this paper, we present the application and procedure of a Sequencing Quality Assessment Tool (SQUAT) featuring pre-assembly and post-assembly analysis. Our tool assists users to examine their sequencing reads from the perspective of base quality scores and alignments against the assembly. We test our tool on six datasets and reveal their sequencing quality through detailed examination. There also exists great potential for further advancement and application, as outlined below.

### Map reads to a reference genome

With regards to species for which a finished genome is available, it is feasible to map sequencing reads to the reference genome for another version of read labeling. As reference-mapping is usually the gold-standard for genome assembly, we can use it as a reference point to examine where the main cause of poor PM% comes from by weighing up the PM% difference between mapping reads to the assemblies and the reference genome. In addition, different paired-end or mate-pair libraries can be mapped to the reference genome to generate reports for comparison.

### Genome assembly with subset selection based on read labels

On top of read label tagging, we also integrate a feature in SQUAT to return a subset of reads containing only certain specified types of reads. For instance, since reads labeled with P and M are usually highly-mapped to assemblies, they may be more ideal for genome assembly. Therefore, this tool can be utilized to implement subset selection suitable for the users.

### Paired-end version and 10X data

So far, only single-end read mapping is available for SQUAT. Namely, no paired-end information is required. In BWA, we can also generate alignments in SAM format given paired-end libraries. However, a different scheme of read label tagging and analysis needs to be designed to fit interleaved read files into the process. In the future, we will place the emphasis on quality assessment of paired-end reads. The current version of SQUAT can perform quality assessment of 10X chromatin-linked reads and the assembly, but it ignores paired-end barcode information. We plan to support paired-end 10X linked reads with barcode analysis.

### Scalability for handling big data of NGS

The scalability issue is important due to the increasingly big data of NGS. The performance bottleneck of SQUAT is the read mapping step despite the step is multi-threading. We think there are two ways to cope with the issue. First, we can use faster read mapping tools, e.g., Kart [25] and minimap2 [26], which are ~ 4 or more times faster than BWA MEM in short read mapping. Second, we can use distributed parallel read mapping tools, e.g., Hadoop-based BigBWA [27] and Spark-based SparkBWA [28]. We plan integrate SQUAT with Kart soon and support distributed parallel in future work.

## Conclusions

SQUAT is an efficient tool for assessing both the quality of sequencing reads and the quality of their genome assembly via read mapping analysis and classification. We carefully defined the poorly mapped (PM) reads into several groups to prevent the underestimation of unmapped reads; indeed, a high PM% would be a sign of a poor assembly that requires researchers’ attention further examination or improvements before using the assembly. We have evaluated SQUAT with six datasets, including eel, worm, mushroom, and three bacteria, and the results show that SQUAT reports provide useful information for assessing the quality of assemblies and reads.

## Additional files

Additional file 1: Pre-assembly reports of the datasets used in Table 3. (ZIP 46 kb)

Additional file 2: The detailed workflow of post-assembly read type labelling. (PDF 498 kb)

Additional file 3: Post-assembly reports of the datasets used in Table 4. (ZIP 3120 kb)

Additional file 4: The details of SQUAT runtime and resource consumption and the results of the wheat dataset. (PDF 364 kb)

## Abbreviations

DQA: Data Quality Assessment
**PM%**: The percentage of Poorly Mapped reads
**SQUAT**: A Sequencing QUality Assessment Tool for data quality assessments before and after Genome Assemblies
