# Supplementary material for: SQUAT: a Sequencing Quality Assessment Tool for data quality assessments of genome assemblies
Source: BMC Genomics. 2019 Apr 18;19(Suppl 9):238. doi: 10.1186/s12864-019-5445-3 (PMC7402383; doi:10.1186/s12864-019-5445-3)
Supplement: Supplementary file 1 — Pre-assembly reports of the datasets used in Table 3. (ZIP 46 kb) [file 12864_2019_5445_MOESM1_ESM.zip › Pre-assembly reports/D1_RMT_ecv_pre_report.htm]

Pre-assembly SQUAT report


Pre-Assembly SQUAT Report

Wed Apr 25 17:40:06 2018

## Summary

Basic Statistics

Overall Categorization

Attributes of FASTQ

Alphabet Frequency & GC content

Quality Statistics

Distribution of Bases' Quality Values

Distribution of Reads' MinimaQ Values

Covergae of Reads with Sufficient High-Quality Bases

  
  
  
  
  
  
Notes: The report requires Internet connection to show the interactive charts of distribuions made by Google chart.

Top
  

# D1\_RMT\_ecv Attributes of FASTQ | Name | Value | | --- | --- | | InputFile | D1\_RMT\_ecv.fastq | | #Read | 1,000,000 | | #Base | 245,196,258 | | AvgReadLen | 245.20 | | MinReadLen | 25 | | MaxReadLen | 251 | Alphabet Frequency & GC content | Name | Count | Freq% | | --- | --- | --- | | A | 36,161,507 | 14.75% | | C | 82,689,422 | 33.72% | | G | 84,344,577 | 34.40% | | T | 40,848,900 | 16.66% | | N | 1,151,852 | 0.47% | | GC% | - | 68.12% | | | | --- | | | Distribution of Bases' Quality Values | Name | AreaFreq | | --- | --- | | Q30 & above | 40.4% | | Q20-Q29 | 8.6% | | Q15-Q19 | 1.7% | | < Q15 | 49.4% | | | | --- | | | Distribution of Reads' MinimalQ Values | Name | AreaFreq | | --- | --- | | % of reads whose bases are all Q20 & above | 1.5% | | % of reads whose bases are all Q15 & above | 2.0% | | % of reads whose bases are all Q10 & above | 2.3% | | | | --- | | | Coverage of Reads with Sufficient High-Quality Bases | Name | Coverage of reads | Remark | | --- | --- | --- | | **% of High-quality reads** Coverage of reads that 100% of their bases with Q20 & above | 1.5% | %HighQ(20) >= 100% (i.e., MinimaQ>=20) | | Coverage of reads that >= 95% of their bases with Q20 & above | 2.9% | %HighQ(20) >= 95% | | Coverage of reads that >= 90% of their bases with Q20 & above | 3.5% | %HighQ(20) >= 90% | | Coverage of reads that >= 90% of their bases with Q15 & above | 3.9% | %HighQ(15) >= 90% | | **% of Poor-quality reads** Coverage of reads that > 10% of their bases with Q14 & less | 96.1% | 1 - {%HighQ(15) >= 90%} | | | | --- | | | | |
