# Supplementary material for: SQUAT: a Sequencing Quality Assessment Tool for data quality assessments of genome assemblies
Source: BMC Genomics. 2019 Apr 18;19(Suppl 9):238. doi: 10.1186/s12864-019-5445-3 (PMC7402383; doi:10.1186/s12864-019-5445-3)
Supplement: Supplementary file 1 — Pre-assembly reports of the datasets used in Table 3. (ZIP 46 kb) [file 12864_2019_5445_MOESM1_ESM.zip › Pre-assembly reports/worm_PE620_pre_report.htm]

Pre-assembly SQUAT report


Pre-Assembly SQUAT Report

Wed Apr 25 19:48:21 2018

## Summary

Basic Statistics

Overall Categorization

Attributes of FASTQ

Alphabet Frequency & GC content

Quality Statistics

Distribution of Bases' Quality Values

Distribution of Reads' MinimaQ Values

Covergae of Reads with Sufficient High-Quality Bases

  
  
  
  
  
  
Notes: The report requires Internet connection to show the interactive charts of distribuions made by Google chart.

Top
  

# worm\_PE620 Attributes of FASTQ | Name | Value | | --- | --- | | InputFile | worm\_PE620.fastq | | #Read | 1,000,000 | | #Base | 251,909,351 | | AvgReadLen | 251.91 | | MinReadLen | 35 | | MaxReadLen | 301 | Alphabet Frequency & GC content | Name | Count | Freq% | | --- | --- | --- | | A | 85,858,977 | 34.08% | | C | 40,082,676 | 15.91% | | G | 39,703,922 | 15.76% | | T | 86,257,285 | 34.24% | | N | 6,491 | 0.00% | | GC% | - | 31.67% | | | | --- | | | Distribution of Bases' Quality Values | Name | AreaFreq | | --- | --- | | Q30 & above | 87.6% | | Q20-Q29 | 5.2% | | Q15-Q19 | 3.8% | | < Q15 | 3.4% | | | | --- | | | Distribution of Reads' MinimalQ Values | Name | AreaFreq | | --- | --- | | % of reads whose bases are all Q20 & above | 3.2% | | % of reads whose bases are all Q15 & above | 6.9% | | % of reads whose bases are all Q10 & above | 99.8% | | | | --- | | | Coverage of Reads with Sufficient High-Quality Bases | Name | Coverage of reads | Remark | | --- | --- | --- | | **% of High-quality reads** Coverage of reads that 100% of their bases with Q20 & above | 3.2% | %HighQ(20) >= 100% (i.e., MinimaQ>=20) | | Coverage of reads that >= 95% of their bases with Q20 & above | 57.7% | %HighQ(20) >= 95% | | Coverage of reads that >= 90% of their bases with Q20 & above | 77.6% | %HighQ(20) >= 90% | | Coverage of reads that >= 90% of their bases with Q15 & above | 96.9% | %HighQ(15) >= 90% | | **% of Poor-quality reads** Coverage of reads that > 10% of their bases with Q14 & less | 3.1% | 1 - {%HighQ(15) >= 90%} | | | | --- | | | | |
