# Supplementary material for: SQUAT: a Sequencing Quality Assessment Tool for data quality assessments of genome assemblies
Source: BMC Genomics. 2019 Apr 18;19(Suppl 9):238. doi: 10.1186/s12864-019-5445-3 (PMC7402383; doi:10.1186/s12864-019-5445-3)
Supplement: Supplementary file 2 — The detailed workflow of post-assembly read type labelling. (PDF 498 kb) [file 12864_2019_5445_MOESM2_ESM.pdf]

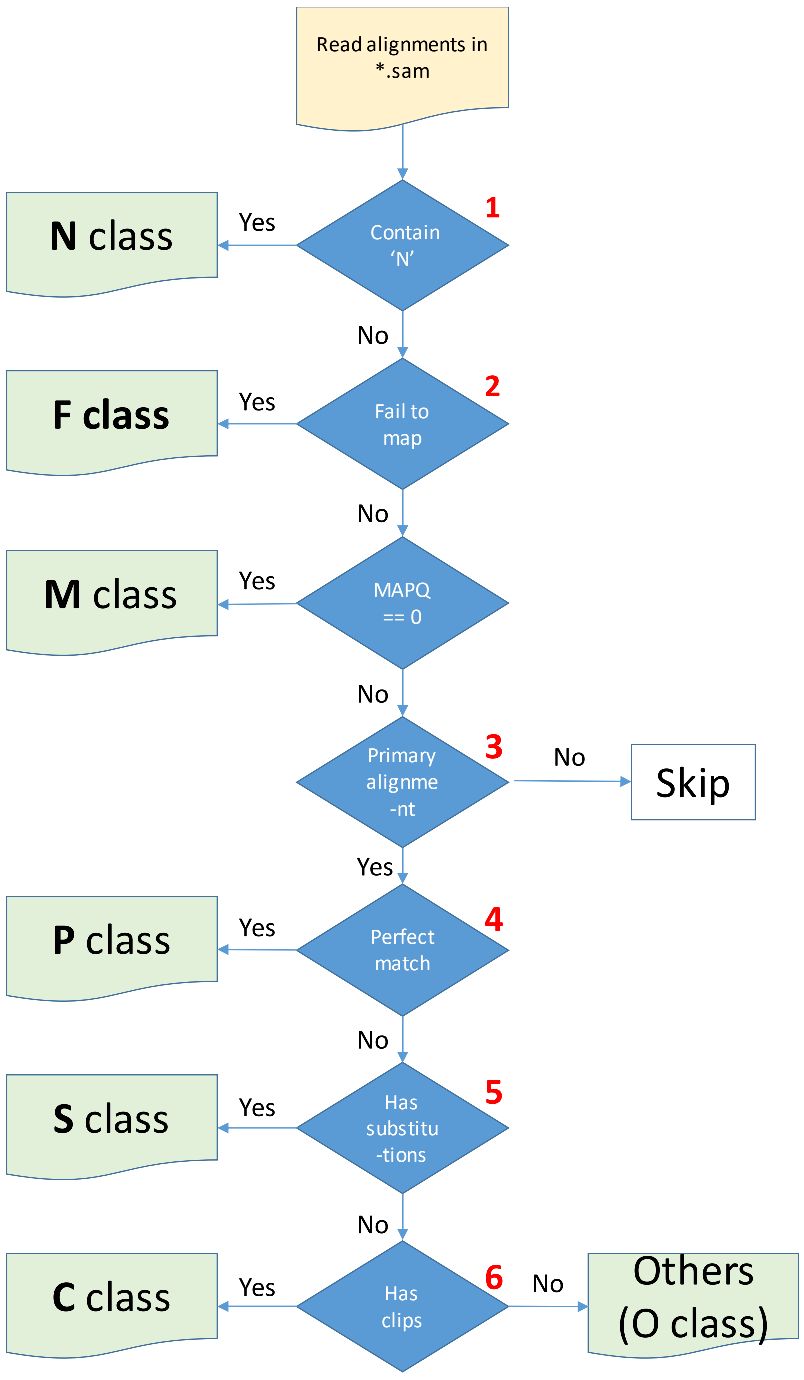

Additional file 2 – The detailed workflow of post-assembly read type labelling

[1] Check if read sequences contain 'N'
[2] Check if field Flag = 0x4
[3] Check if field Flag != 0x100 and Flag != 0x800 (not secondary & supplementary alignments)
[4] Check if field CIGAR has only 'M' operation and perfect match
[5] Check if field CIGAR has only 'M' operation and at least one mismatch (the NM tag)
[6] Check if field CIGAR has 'S' operation
