# Supplementary material for: SQUAT: a Sequencing Quality Assessment Tool for data quality assessments of genome assemblies
Source: BMC Genomics. 2019 Apr 18;19(Suppl 9):238. doi: 10.1186/s12864-019-5445-3 (PMC7402383; doi:10.1186/s12864-019-5445-3)
Supplement: Supplementary file 3 — Post-assembly reports of the datasets used in Table 4. (ZIP 3120 kb) [file 12864_2019_5445_MOESM3_ESM.zip › Post-assembly reports/D2_MMT_ecv_post_report.html]

Post-assembly report


Post-Assembly SQUAT Report

2018-04-25 17:45

## Summary

Basic Statistics

Label Distrbution (Table)

Label Distribution (Barchart)

BWA - MEM (local)

Mismatch ratio of S reads

Clip ratio of C reads

Alignment score of P reads

Alignment score of S reads

Alignment score of C reads

BWA - backtrack (end2end)

Alignment score of S reads

Clip ratio of C reads

|  |  |
| --- | --- |
| **Label** | **Description** |
| P | Perfectly-matched reads |
| S | Reads with substitution errors |
| C | Reads containing clips |
| O | Reads with other errors |
| M | Multi-mapped reads |
| F | Unmapped reads |
| N | Reads containing N |

|  |  |
| --- | --- |
| **Threshold** | **Value** |
| Overall PM% | 20% |
| Type S | 20% |
| Type C | 30% |
| Type O | 10% |
| Type N | 10% |

Top

## D2\_MMT\_ecv

### Percentage of poorly-mapped reads (PM%): 11.4%

*PM% - type F: 8.5%**PM% - type C: 2.9%*

## Basic Statistics

| Seqeuencing reads | Value |
| --- | --- |
| File name | D2\_MMT\_ecv.fastq |
| No. of sequence | 8,739,133 |
| Sample size | 1,000,000 |
| Sequence length | 25 - 251 |
| Avg. poorly mapped sequence% | 11.4% |
| GC% | 64% |

| Reference assembly | Value |
| --- | --- |
| File name | scaffolds.fasta |
| # Scaffolds | 912 |
| Assembly Size (Mbp) | 5.6 |
| Max Scaffolds (Kbp) | 1,346.8 |
| N25 (Kbp) | 481.4 |
| N50 (Kbp) | 313.9 |
| N75 (Kbp) | 144.7 |
| L80 | 14 |
| L90 | 23 |
| L99 | 765 |
| # N's per 100 kbp | 0.00 |
| GC (%) | 61.65 |

## Label Distribution Table

|  | | BWA MEM | BWA backtrack |
| --- | --- | --- | --- |
| Uniquely-mapped Reads | P | 54.5% | 54.5% |
|  | S | 28.4% | 28.9% |
|  | C | 16.0% | 0.0% |
|  | O | 0.1% | 0.2% |
| M | | 0.0% | 0.0% |
| F | | 0.8% | 16.1% |
| N | | 0.2% | 0.2% |

## Label Distribution Barchart

## BWA - MEM

## BWA - backtrack
