# Supplementary material for: SQUAT: a Sequencing Quality Assessment Tool for data quality assessments of genome assemblies
Source: BMC Genomics. 2019 Apr 18;19(Suppl 9):238. doi: 10.1186/s12864-019-5445-3 (PMC7402383; doi:10.1186/s12864-019-5445-3)
Supplement: Supplementary file 4 — The details of SQUAT runtime and resource consumption and the results of the wheat dataset. (PDF 364 kb) [file 12864_2019_5445_MOESM4_ESM.pdf]

## Additional file 4 – The details of SQUAT runtime and resource consumption and the results of the wheat dataset

**Table S1. SQUAT runtime and resource consumption of the D1 dataset**

| D1: #sampled_reads | 1M | 2M | 5M |
|---|---|---|---|
| User time (seconds) | 1146.66 | 2256.12 | 5381.74 |
| System time (seconds) | 68.36 | 125.46 | 375.08 |
| Percent of CPU this job got | 160% | 152% | 179% |
| Elapsed (wall clock) time (h:mm:ss or m:ss) | 12:38.2 | 25:56.9 | 53:19.8 |
| Maximum resident set size (kbytes) | 2,194,768 | 4,263,856 | 10,759,928 |
| File system inputs | 3,204,496 | 6,420,536 | 16,442,864 |
| File system outputs | 4,763,120 | 9,460,984 | 23,554,192 |

**Table S2. SQUAT runtime and resource consumption of the D2 dataset**

| D2: #sampled_reads | 1M | 2M | 5M |
|---|---|---|---|
| User time (seconds) | 733.51 | 727.5 | 761.75 |
| System time (seconds) | 79.58 | 80.66 | 57.85 |
| Percent of CPU this job got | 61% | 58% | 59% |
| Elapsed (wall clock) time (h:mm:ss or m:ss) | 22:03.5 | 23:05.3 | 22:53.7 |
| Maximum resident set size (kbytes) | 2,324,264 | 2,324,528 | 2,324,984 |
| File system inputs | 3,587,720 | 4,358,944 | 5,951,296 |
| File system outputs | 4,231,776 | 4,232,880 | 4,235,496 |

**Table S3. SQUAT runtime and resource consumption of the wheat dataset**

| Wheat library | SRR5815659_1 | SRR5815659_2 |
|---|---|---|
| Read length (bp) | 150 | 150 |
| #read | 394,218,444 | 394,218,444 |
| #sampled_read | 1,000,000 | 1,000,000 |
| Machine | CPU: Intel Xeon E7-4830 v3 2.1GHz, 48 cores (96 hyperthreads); 1.5TB RAM | Vmware VM, 8 cores, 128G RAM (Machine: Intel Xeon E7-4820 2GHz, 32 cores (64 hyperthreads), 128GB RAM) |
| Peak CPU usage (hyperthreads) | 32 | 8 |
| Elapsed (wall clock) time (h:mm:ss) | 09:52:48 | 20:33:56 |
| Peak memory usage (KB) | 14,269,024 | 11,401,808 |
| KB read per second | 117,621.87 | 63,005.16 |
| KB write per second | 135,184.93 | 93,534.40 |

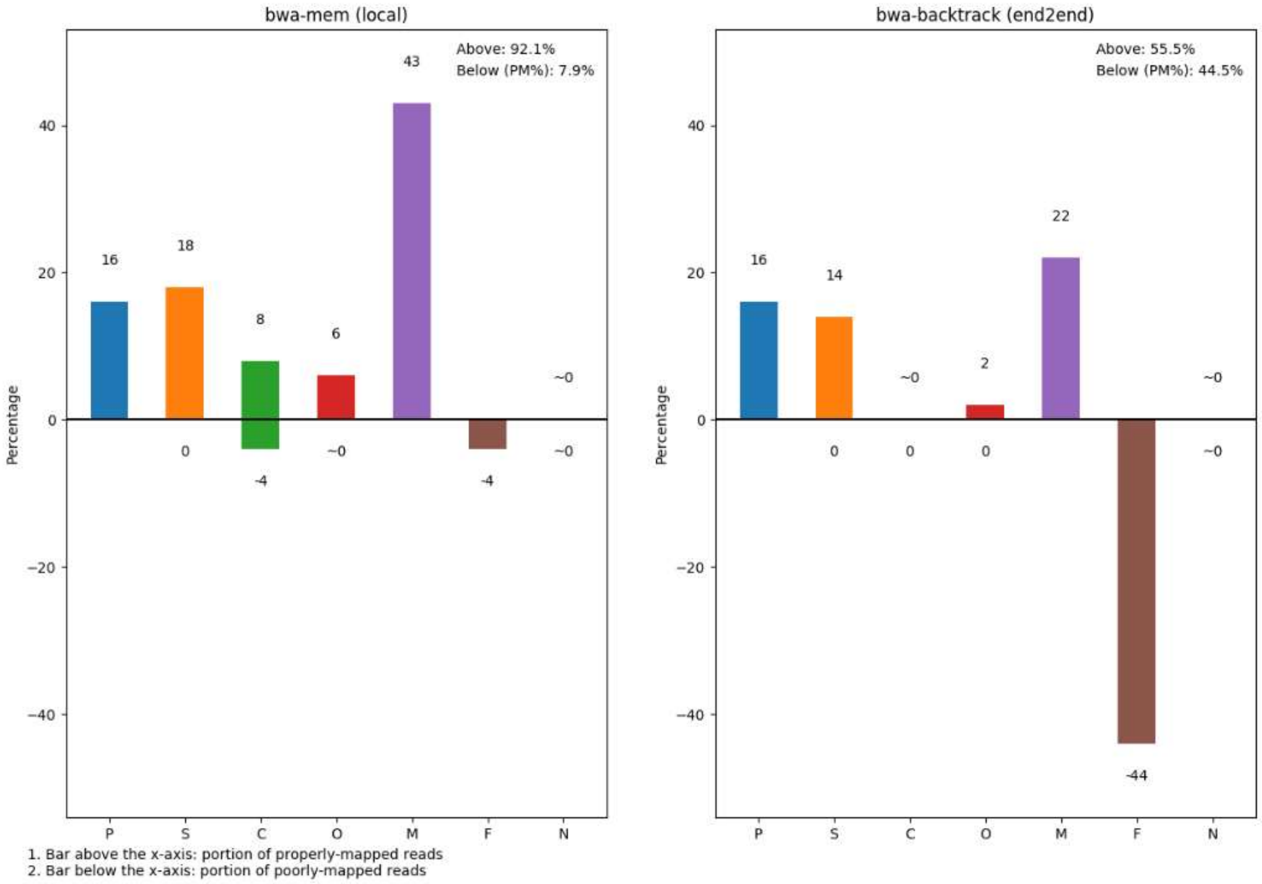


**Figure S1. Post-assembly label distribution barchart of the wheat SRR5815659_1 dataset**

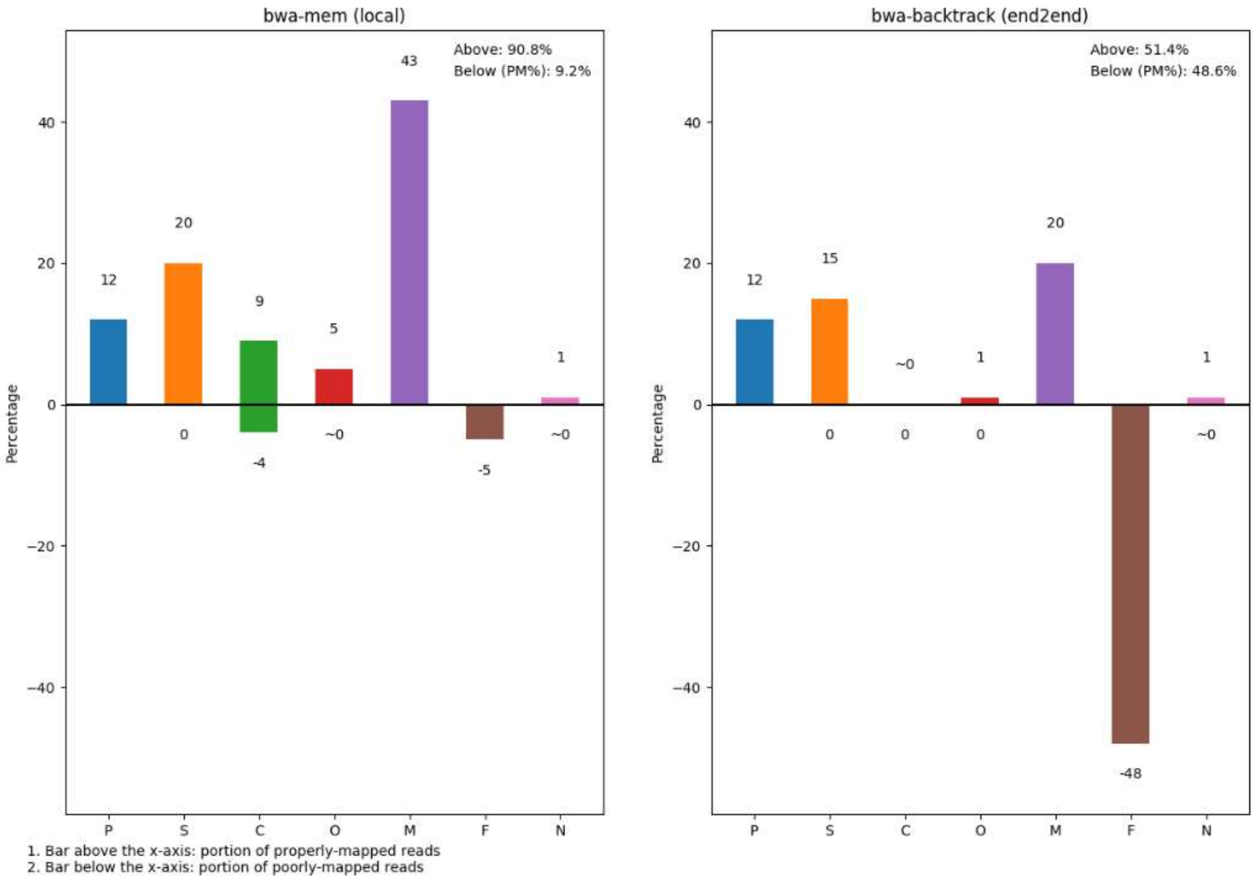


**Figure S2. Post-assembly label distribution barchart of the wheat SRR5815659_2 dataset**
